# Novel compound heterozygous mutations of the *NPC1* gene associated with Niemann-pick disease type C: a case report and review of the literature

**DOI:** 10.1186/s12879-024-09025-5

**Published:** 2024-01-30

**Authors:** Chaoxin Tao, Min Zhao, Xiaohui Zhang, Jihong Hao, Qiuyue Huo, Jie Sun, Jiangtao Xing, Yuna Zhang, Jianhong Zhao, Huaipeng Huang

**Affiliations:** 1 Department of Internal Medicine, Shijiazhuang Ping’an Hospital, Hebei Medical University, Shijiazhuang, Hebei China; 2 Hebei Provincial Center for Clinical Laboratories, The Second Hospital of Hebei Medical University, Shijiazhuang, Hebei China; 3 Department of Clinical Laboratory, The Second Hospital of Hebei Medical University, Shijiazhuang, Hebei China; 4 Department of Ultrasound Diagnosis of Gynecology and Obstetrics, The Second Hospital of Hebei Medical University, Shijiazhuang, Hebei China

**Keywords:** Niemann-pick disease, Compound heterozygous mutations, *NPC1* gene, Novel mutation, Case report

## Abstract

**Background:**

Niemann-Pick Disease type C is a fatal autosomal recessive lipid storage disorder caused by *NPC1* or *NPC2* gene mutations and characterized by progressive, disabling neurological deterioration and hepatosplenomegaly. Herein, we identified a novel compound heterozygous mutations of the *NPC1* gene in a Chinese pedigree.

**Case presentation:**

This paper describes an 11-year-old boy with aggravated walking instability and slurring of speech who presented as Niemann-Pick Disease type C. He had the maternally inherited c.3452 C > T (p. Ala1151Val) mutation and the paternally inherited c.3557G > A (p. Arg1186His) mutation using next-generation sequencing. The c.3452 C > T (p. Ala1151Val) mutation has not previously been reported.

**Conclusions:**

This study predicted that the c.3452 C > T (p. Ala1151Val) mutation is pathogenic. This data enriches the *NPC1* gene variation spectrum and provides a basis for familial genetic counseling and prenatal diagnosis.

## Background

Niemann-Pick Disease (NPD) is a rare autosomal recessive disorder that is characterized by progressive neurodegenerative disease. Most patients have liver, kidney, brain, bone marrow and other organ lesions due to excessive accumulation of sphingomyelin in cells of the monocyte/macrophage system and other tissues. This accumulation is a result of the lack of sphingomyelinase. Based on pathogenesis and pathogenic genes, NPD is divided into three types: A, B and C [1]. Niemann-Pick Disease types A and B are allelic disorders caused by mutations in the sphingomyelin phosphodiesterase-1 (*SMPD1*) gene [2]. Niemann-Pick Disease type C (NPC) is an autosomal recessive lipid storage disorder that is characterized by a cholesterol transport disorder and progressive neurodegeneration. Niemann-Pick Disease type C is divided into type C1 and type C2, which are caused by pathogenic mutations of the *NPC1* gene and *NPC2* gene, respectively [3, 4]. Niemann-Pick Disease type C most commonly affects the nervous system, with clinical manifestations that include hypotonia, motor retardation, vertical supranuclear gaze palsy (VSGP) and cataplexy. The identification of two copies of known disease-causing mutations in either *NPC1* or *NPC2* gene, where the mutations are on opposite chromosomes confirms the diagnosis of NPC [5]. According to a consensus clinical management guideline for NPC, approximately 700 *NPC1* gene variants have been reported, of which approximately 420 are considered to be pathogenic. There are only a limited number of common (p. I1061T, p. P1007A) or recurrent (p. R978C, p. G992R, p. D874V) mutations [6]. The I1061T site is a hot spot for the mutation in western European countries [7]. Thus, the interpretation of new missense and splicing mutations should be undertaken with caution and their pathogenic nature must be verified. Here, we described an 11-year-old Chinese boy with NPC.

## Case presentation

An 11-year-old boy was admitted to Shijiazhuang Ping’an Hospital, Hebei Medical University, because of aggravated walking instability and slurring of speech. He had been hospitalized in a local hospital for 10 days due to neonatal hyperbilirubinemia and hepatosplenomegaly at birth. He was then hospitalized to treat liver and spleen enlargement and thrombocytopenia at the age of four months but failed to recover. He was prone to nasal bleeding, which was difficult to stop.

**Fig. 1:**
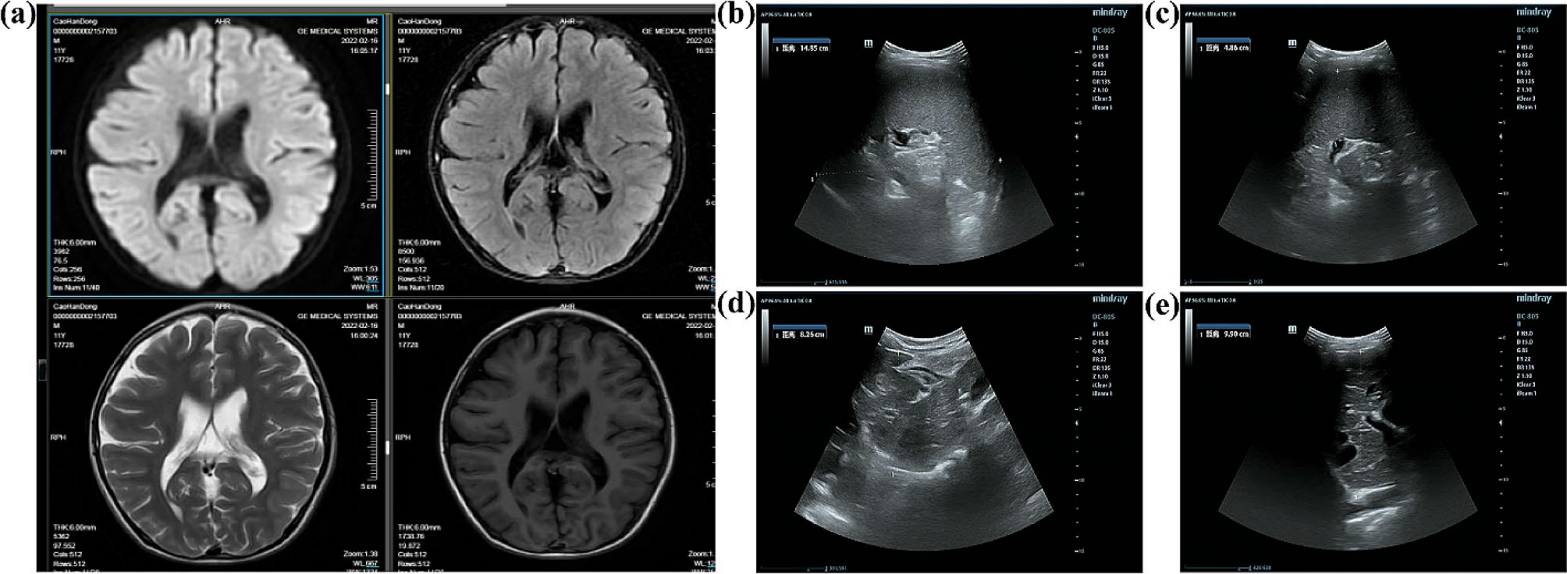
Brain CT and abdominal ultrasound of the patient. **(a)** A CT scan of the patient’s brain showed a slight widening of the sulcal fissure. **(b, c)** Length and thickness of the patient’s spleen. **(d, e)** Anteroposterior diameter of the left and right lobes of the liver

**Fig. 2:**
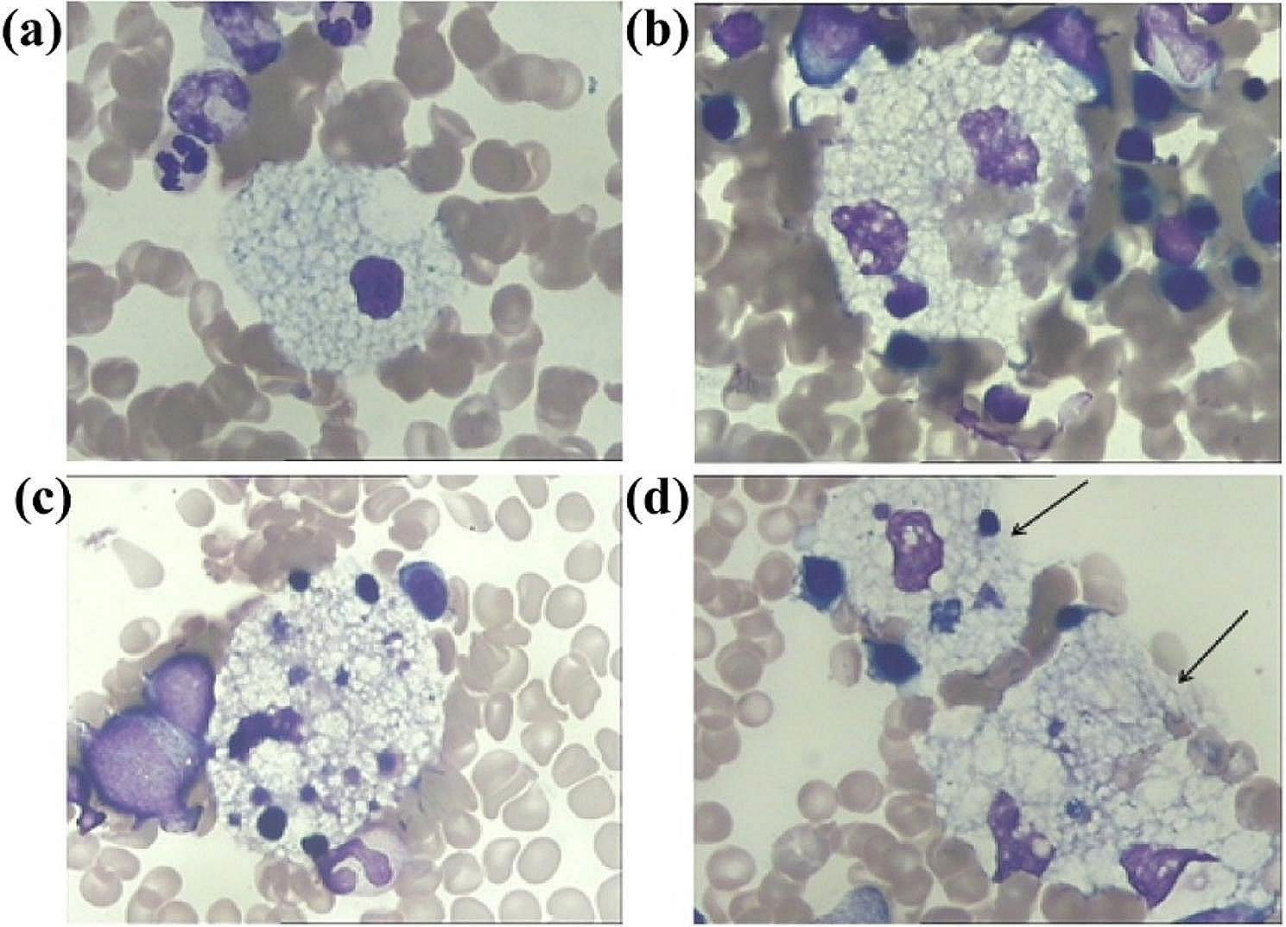
Bone marrow smear of the patient. **(a)** Mononuclear Niemann-Pick cells. **(b)** Binucleate Niemann-Pick cells. **(c, d)** Niemann-Pick cells phagocytosing blood cells in the bone marrow smear. (Wright-Giemsa staining ×1000)

A physical examination revealed that he could not touch the ground with his heel when squatting and he found it difficult to rise from a squat. He had vertical gaze palsy and limited up and down movement of both eyes. The neurological examination showed that the dysdiadochokinesia, VSGP, plus the finger-nose test and the eyes closed difficult to stand sign was positive. The blood examination showed elevated levels of serum aspartate aminotransferase (50.5U/L) and triglycerides (2.01mmol/L). A brain MRI showed slightly widened ventricles and sulcus (Fig. 1a). A chest radiograph showed slightly more texture than is normal in both lungs. An abdominal ultrasound showed that the spleen was 4.8 cm thick (standard range: 7–12 years old < 4.0 cm) and 15.4 cm long (standard range: 8–12 years old < 11.5 cm) (Fig. 1b and c). The anteroposterior diameter of the right lobe of the liver was 9.9 cm, whilst the anteroposterior diameter of the left lobe was 8.3 cm (standard range: 9–12 years old < 9.1 cm) (Fig. 1d and e). When the clinical manifestations and medical history were combined, the child was suspected of having NPD. Foamy cells were observed on the bone marrow smears. These cells were large, round or oval in shape and had abundant cytoplasm. The nuclei were located in the center or to one side of the cells. The cytoplasm was filled with vacuoles (Fig. 2a and b) and erythrophagocytosis was observed on the smear (Fig. 2c and d).

**Fig. 3:**
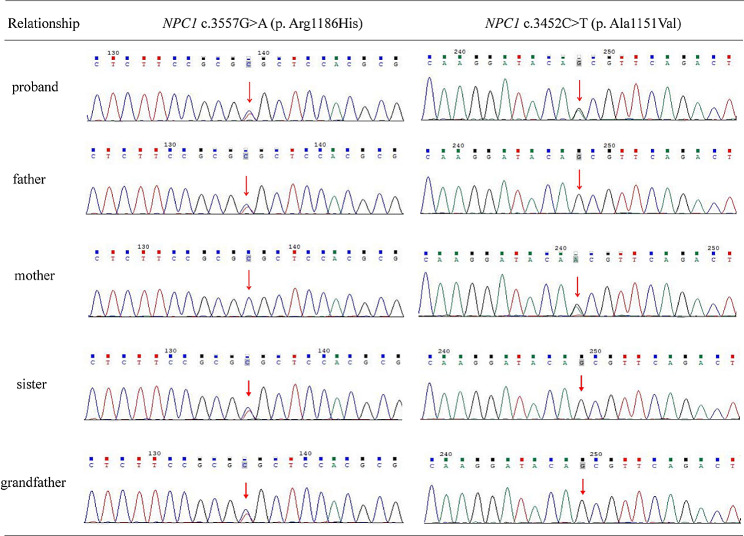
Gene sequencing validation results for the patient, parents, sister and grandfather

Next-generation sequencing revealed two mutation sites in the *NPC1* gene of the proband. One chromosome had the c.3452 C > T (p. Ala1151Val) missense mutation in exon 22, inherited from his mother and not previously reported. The other chromosome had the c.3557G > A (p. Arg1186His) mutation in exon 23, inherited from his father (Fig. 3). Based on the above findings, the patient was confirmed as having NPC and was treated with Miglustat to reduce the accumulation of neurotoxicity. The treatment was also used to stabilize and improve neurological symptoms and prevent or delay disease progression. Since the diagnosis, the patient has had regular bimonthly follow-ups with normal blood counts and liver and kidney function results, but hepatosplenomegaly persisted.

## Discussion

Here, we reported the case of a child diagnosed with NPC, who was born with persistent jaundice and hepatosplenomegaly, but had a normal neurodevelopment. In the previous six months (11 years old), the patient had presented with neurological symptoms such as poor speech, walking instability, falling easily and learning disabilities. A large number of typical Niemann-Pick cells were seen on a bone marrow smear. Genetic testing of the child revealed two mutation sites in the *NPC1* gene; the c.3557G > A (p. Arg1186His) heterozygous variant and the c.3452 C > T (p. Ala1151Val) heterozygous variant. The two variants created a compound heterozygous mutation (Table 1). Using all the examinations and clinical manifestations, this child was diagnosed with NPC.

**Table 1:** *NPC1* mutation sites in the patient, parents, sister and grandfather

Gene	Chromosomal Location (hg19)	HGVS Name	Gene Subregion	Type of Mutation	Zygotic State					ACM Rating
					Proband	Father	Mother	Sister	Grandfather	
*NPC1*	chr18:21114444	NM_000271.5: c.3557G > A (p. Arg1186His)	Exon 23	missense mutation	heterozygote	heterozygote	wild type	heterozygote	heterozygote	pathogenic
*NPC1*	chr18:21115458	NM_000271.5: c.3452 C > T (p. Ala1151Val)	Exon 22	missense mutation	heterozygote	heterozygote	heterozygote	wild type	wild type	May cause disease

Niemann-Pick Disease type C is a rare, autosomal recessive lysosomal lipid storage disease that is characterized by progressive neurodegeneration. Cholesterol transport disorders are caused by mutations in *NPC1* gene (95% of cases), on chromosome 18, and/or *NPC2* gene (5% of cases), on chromosome 14. They are characterized by decreased self-renewal and neurodegeneration of neural stem cells [8, 9], with an incidence of 1 in 150,000 to 1 in 100,000 [10, 11]. *NPC1* and/or *NPC2* gene causes altered calcium homeostasis, leading to the secondary storage of sphingolipids and cholesterol. Our patients also showed abnormal cholesterol. Hence, plasma cholesterol can be determined as a useful biomarker. The *NPC1* gene is located on chromosome 18q11-q12 and has 25 exons that encode a membrane protein of 1278 amino acids. More than 400 *NPC1* gene mutations have been described and include missense, nonsense variants, splicing mutations, deletions and insertions [12]. Due to the atypical clinical manifestations in the early stage of NPC, it is easily confused with other lysosomal lipid storage diseases or metabolic diseases, which increases the difficulty of diagnosis. However, genetic testing can provide strong evidence for the diagnosis of NPC.

In this study, two missense mutations, c.3557G > A (p. Arg1186His) and c.3452 C > T (p. Ala1151Val), were found in the *NPC1* gene. The c.3557G > A (p. Arg1186His) heterozygous mutation is a previously reported pathogenic mutation, it is a missense mutation located in the 6th small cytoplasmic loop, between the TM 11 and 12 domains of the NPC1 protein, altering an amino acid that is phylogenetically conserved. This mutation may result in mislocalization of intracellular proteins [12]. The c.3452 C > T (p. Ala1151Val) is a new mutation that is not found in the GnomAD database, ExAC database or 1000 Genomes database. It has also not been observed in NPC patients. We used REVEL, BayesDel and MutPred models to predict the function of the mutation site. The results indicated that the mutation is harmful. The c.3451G > A (p. Ala1151Thr) mutation is located at the same amino acid site as the c.3452 C > T (p. Ala1151Val) mutation and has been detected in multiple NPC patients. The ClinVar database classified the c.3451G > A (p. Ala1151Thr) mutation as a possible pathogenic mutation. In accordance with the American College of Medical Genetics rating rules, the c.3452 C > T (p. Ala1151Val) mutation was classified as a possible pathogenic mutation, which will be verified later. Costanzo et al. [13] reported the case of a woman with a compound heterozygous mutation in *NPC1* gene. The clinical manifestations and age of onset of this woman were similar to our patient. Both of them showed neurological symptoms such as learning disabilities and unclear speech. However, this woman came to the clinic at the age of 38. Völkner et al. [14] also described a female patient who had a compound heterozygous mutation that included a point mutation, p. G992R (c.2974G > C), on one allele and a frameshift mutation, p. V1023Sfs *15 (c.3066_3073d), on the other allele. This resulted in premature protein truncation. The patient appeared normal before the age of 20 and gradually developed neurological symptoms at the age of 24. Studies have reported that the p. G992R mutation is associated with the late onset of NPC [15]. Therefore, different mutation sites and mutation types of the *NPC1* gene may lead to large differences in age of onset and clinical manifestations. Furthermore, we pooled information on patients with NPC with compound heterozygous mutations (Table 2). The results showed that phenotypic differences also existed between patients carrying the same mutation type even within the same family, it is not exactly known whether phenotype is based on specific *NPC1* gene mutations or whether other aspects of the patient′s genetic background play a role. Further studies are needed to understand whether the type of mutation affects the clinical presentation of patients.

**Table 2:** Summary of cases of Niemann-Pick disease type C presenting with compound heterozygous mutations

Case	Sex	Age (years)	onset of neurological signs (years)	Onset of neurological signs	Outcome	Nucleotide mutation	Amino acid change		Reference
**Substitution**									
1	F	51	None^**b**^	Hepatosplenomegaly, Proteinuria, Lamellar bodies (LB)	Alive	c.2474 A > G c.1301 C > T		p. Tyr825Cys p. Pro434Leu	Pintavorn, P (2022) [16]
2	F	38	28	Dysmetria, Ataxia, Cerebellar atrophy	Alive	c.1553G > A c.1270 C > T		p. Arg518Gln p. Pro424Ser	Costanzo, M. C (2020) [13]
3	M	6	6	Hepatocellular, carcinoma, Hypoglycemia, Abdominal distension, Hepatosplenomegaly	Died	c.338G > A c.2780 C > T		p. C113Y p. A927V	Hwang, S (2022) [17]
4	F	2	-	Visceral Enlargement, Cholestasis, Neurological involvement	-	c.2830G > A c.3104 C > T		-	López de Frutos, L (2020) [18]
5	M	27	23	Dysarthria, Dysphagia,	Alive	-		p. Ser954Leu p. Asn1156Ser	Maubert, A (2015) [19]
6	F	13	12	Ataxia, Cerebellar atrophy, Epilepsy, Dysphagia, Dysarthria	Died	c.2498G > A c.1858 A > T		p. Trp833X p. Ile609Phe	Fusco, C (2013) [20]
7	M	12	7	Dyspraxia, Cerebellar abnormalities, Splenomegaly	Alive	c.1955 C > G c.2107T > A		p. Ser652Trp p. Phe703Ile	Soliani, L (2020) [21]
8	M	9	4	Dysarthria, Hepatosplenomegaly, Vertical saccades	Alive	c.1955 C > G c.2107T > A		p. Ser652Trp p. Phe703Ile	Soliani, L (2020) [21]
9	M	26 days	None	Hepatosplenomegaly	Alive	c.2728G > A c.269 C > G		p. G910S p. P90R	Zhang, G (2019) [22]
10	M	25	11	Splenomegaly, Dysphagia, Ataxia, VSGP	Alive	c.1421 C > T c.3722 T > C		p. P474L p. L1241S	Kawazoe, T (2018) [23]
11	F	9	3	Hepatosplenomegaly, Brain atrophy	Died	c.2108T > C c.2348 C > G		p. F703S p. S813X	Kodachi, T (2017) [24]
12	F	66	None	Splenomegaly	Alive	c.1133T > C c.1990G > A		p. V378A p. V664M	Greenberg, C. R (2015) [25]
13	M	24	21	Ataxia, VSGP, Generalized dystonia, Dysarthria, Brain atrophy	Alive	c.1552 C > T c.2780 C > T		p. R518W p. A927V	Lee, S. Y (2016) [26]
14	F	23	19	Ataxia, VSGP, Hepatosplenomegaly, Diffuse brain atrophy	Alive	c.1552 C > T c.2780 C > T		p. R518W p. A927V	Lee, S. Y (2016) [26]
15	M	19	9	Ataxia, Dysarthria, VSGP, Splenomegaly	Died	c.1042 C > T c.2780 C > T		p. Arg348* p. Ala927Val	Cervera-Gaviria, M (2016) [27]
**Deletion-insertion**									
16	F	33	24	Ataxia, Dysarthria, Dysphagia, VSGP^**a**^, Hepatosplenomegaly	Alive	c.3066_3073delinsT c.2974G > C		p. V1023Sfs*15 p. G992R	Völkner, C (2021) [14]
17	M	2	-	Visceral Enlargement	-	c.2882_2897delinsG c.2974G > T		-	López de Frutos, L (2020) [18]
**Insertion**									
18	F	12	None	Hepatosplenomegaly	Alive	c.3591 + 3insT c.3170 A > C		IVS23 + 3insT p. K1057R	Bountouvi, E (2017) [28]
19	F	28	27	Splenomegaly, Painful dystonia	Alive	c.3011 C > T c.160_161insG		p. S1004L p. D54GfsX4	Kawazoe, T (2018) [23]
20	F	35	22	Splenomegaly, Schizophrenia	Alive	c.3011 C > T c.160_161insG		p. S1004L p. D54GfsX4	Kawazoe, T (2018) [23]
**Deletion**									
21	M	1-years 10-months	-	Visceral Enlargement, Neurological involvement	-	c.1757delA`a c.2746_2748delAAT		-	López de Frutos, L (2020) [18]
22	F	2-months	-	Visceral Enlargement	-	c.352_353delAG c.530G > A		-	López de Frutos, L (2020) [18]
23	M	4	2-years 6-months	Dysphagia, Ataxia, VSGP	Died	c.864delT c.3618delA		p. F288LfsX22 p. K1206NfsX36	Kumagai, T (2019) [29]
24	F	infant	None	Hepatosplenomegaly	Alive	c.395delC c.2068insTCCC		-	Lo, S. M (2010) [30]

VSGP^**a**^: Vertical supranuclear gaze palsy

None^**b**^: Remains free of any neurological or psychiatric manifestations

Individual heterogeneity is high in NPC. The broad clinical spectrum ranges from a neonatal rapidly fatal disorder to an adult-onset chronic neurodegenerative disease. The neurological involvement defines the disease severity in most patients, and typically preceded by systemic signs such as neonatal hyperbilirubinemia, cholestatic jaundice in the neonatal period or hepatosplenomegaly in infancy or childhood. Symptoms and disease course of NPC are significantly influenced by the age at which neurological manifestations occur. In accordance with the 2018 consensus guidelines for clinical management of NPC, the age of onset is divided into five types: neonatal (0–3 months), early infantile period (3 months to 2 years), late infantile period (2 to 6 years), juvenile period (6–15 years) and adult form (> 15 years) [6, 31]. The clinical manifestations and prognosis of each type are significantly different. The neonates show prolonged jaundice, ascites and persistent hepatosplenomegaly. Approximately 10% of children develop liver failure and die within six months due to progressive jaundice. At this stage, the neurological symptoms of children are often not obvious. Early infantile children first show isolated hepatosplenomegaly [32]. Neurological symptoms generally begin to appear from eight to nine months old and the symptoms are more obvious by one to two years old [33]. The overall survival for this type of child rarely exceeds six years. The late infantile children are the first to show hepatosplenomegaly, patients present with early downward gaze palsy, a symptom that is not easily detected. In addition, ataxia, unsteady walking and fall-prone occurs during this period, and may be found to have cognitive deficits that progress insidiously. The survival time for most patients is seven to twelve years. Patients of juvenile type present with learning difficulties, unsteady walking gait, fall easily, most have seizures, and physical examination may reveal splenomegaly, and the survival time for this group is generally approximately 30 years [34]. Adult patients may only show isolated splenomegaly and lack neurological symptoms. The prognosis for this group is also related to the time of neurological symptoms and whether they have associated severe epilepsy. The prognosis for adult patients is better than for other groups. Our patient belongs to the juvenile type and was born with persistent jaundice and hepatosplenomegaly. The patient had a normal neurodevelopment before the age of 11. The suspicion of NPC began when he was 11 years old. The persisting hepatosplenomegaly in association with the neurologic findings including dysdiadochokinesia, VSGP, plus the finger-nose test positive, led to the clinical suspicion of NPC confirmed by next-generation sequencing. Organomegaly, although not a consistent feature, presents as splenomegaly and/or hepatomegaly and may precede the onset of neurologic abnormality for many years.

This case enriches the types of *NPC1* gene mutations, suggesting that we should actively learn the medical and family histories and perform a detailed physical examination in patients with prolonged neonatal jaundice, unexplained hepatosplenomegaly, dyskinesia and neuropsychiatric symptoms. At the same time, by summarizing NPC patients with compound heterozygous mutations, it is shown that NPC patients have a wide range of age of onset, and their clinical symptoms are complex and diverse. Comprehensive clinical evaluation, neurological examination and laboratory test are quite important for the diagnosis of NPC. The role of genetic testing in diagnosing NPC should be emphasized, more attention should be paid to the application of screening and diagnostic tools in the future.

## Conclusions

In summary, we presented a case of an NPC patient in whom the disease was caused by a novel compound heterozygous mutation of the *NPC1* gene. This study has expanded the understanding of the types of *NPC1* gene mutations. For patients with neurological-specific manifestations, such as VSGP, ataxia and learning disabilities, genetic testing should be emphasized. Early diagnosis of the disease can help to improve the prognosis of patients.

## Data Availability

No datasets were generated or analysed during the current study.

## Abbreviations

NPD: Niemann-Pick Disease
NPC: Niemann-Pick Disease type C
VSGP: Vertical supranuclear gaze palsy
